# Kinetics of PKCε Activating and Inhibiting Llama Single Chain Antibodies and Their Effect on PKCε Translocation in HeLa Cells

**DOI:** 10.1371/journal.pone.0035630

**Published:** 2012-04-20

**Authors:** Milla Summanen, Niko Granqvist, Raimo K. Tuominen, Marjo Yliperttula, C. Theo Verrips, Johannes Boonstra, Christophe Blanchetot, Elina Ekokoski

**Affiliations:** 1 Cell Biology, Department of Biology, University of Utrecht, Utrecht, The Netherlands; 2 Division of Pharmacology and Toxicology, Faculty of Pharmacy, University of Helsinki, Helsinki, Finland; 3 Division of Biopharmaceutics and Pharmacokinetics, Faculty of Pharmacy, University of Helsinki, Helsinki, Finland; Blaise Pascal University, France

## Abstract

Dysregulation of PKCε is involved in several serious diseases such as cancer, type II diabetes and Alzheimer's disease. Therefore, specific activators and inhibitors of PKCε hold promise as future therapeutics, in addition to being useful in research into PKCε regulated pathways. We have previously described llama single chain antibodies (VHHs) that specifically activate (A10, C1 and D1) or inhibit (E6 and G8) human recombinant PKCε. Here we report a thorough kinetic analysis of these VHHs. The inhibiting VHHs act as non-competitive inhibitors of PKCε activity, whereas the activating VHHs have several different modes of action, either increasing V_max_ and/or decreasing K_m_ values. We also show that the binding of the VHHs to PKCε is conformation-dependent, rendering the determination of affinities difficult. Apparent affinities are in the micromolar range based on surface plasmon resonance studies. Furthermore, the VHHs have no effect on the activity of rat PKCε nor can they bind the rat form of the protein in immunoprecipitation studies despite the 98% identity between the human and rat PKCε proteins. Finally, we show for the first time that the VHHs can influence PKCε function also in cells, since an activating VHH increases the rate of PKCε translocation in response to PMA in HeLa cells, whereas an inhibiting VHH slows down the translocation. These results give insight into the mechanisms of PKCε activity modulation and highlight the importance of protein conformation on VHH binding.

## Introduction

Protein kinase C (PKC) is a family of serine/threonine kinases that regulate several signaling pathways in cells. The ten PKC isozymes have distinct biological functions and are divided into three groups based on cofactor requirements [1]. All of the PKC isozymes are regulated by phosphatidylserine (PS). In addition, conventional PKCs (α, βI, βII and γ) are activated by Ca^2+^ and diacylglycerol (DAG), novel PKCs (δ, ε, η and θ) require only DAG for activation, and atypical PKCs (ζ and ι/λ) are insensitive to both DAG and Ca^2+^ [2]. Conventional and novel PKC isozymes translocate to the plasma membrane when DAG or its surrogate, phorbol 12-myristate 13-acetate (PMA), which is often used as a PKC activator in cellular assays, become available [3]. In addition to cofactor binding, PKC activity is also regulated by priming phosphorylations of three conserved phosphorylation motifs [1] and protein-protein interactions such as binding to receptors for activated C kinase (RACKs) [4].

PKCε plays essential roles in a variety of signaling systems including those regulating proliferation, differentiation, gene expression, metabolism, transport, and muscle contraction [5]. Therefore, it is not surprising that its dysregulation is implicated as a player in several serious diseases including cancer [6], [7], diabetes mellitus [8], [9] and Alzheimer's disease [10].

In cancer, PKCε is considered a transforming oncogene that can contribute to malignancy either by enhancing cell proliferation or by inhibiting cell death [6]. PKCε has been found to be overexpressed in tumor-derived cell lines and in tumor specimens from various organ sites, and is considered to be the PKC isozyme with the greatest oncogenic potential [11]. Furthermore, *in vitro* studies have shown that overexpression of PKCε increases proliferation, motility and invasion of fibroblasts or immortalized epithelial cell lines [7]. One of the mechanisms by which PKCε controls cell division is through its role in cytokinesis. PKCε associates with 14-3-3 scaffold proteins to regulate abscission, a process which requires PKCε kinase activity [12].

In type II diabetes, PKCε has been identified as one of the proteins involved in insulin resistance [13]. Activated PKCε reduces the insulin receptor (IR) gene promoter activation, decreasing the number of IR's on the cell surface, thereby leading to a decrease in insulin sensitivity [8]. The decrease in IR numbers on the cell surface is mediated by the transcription factor HMGA1, which is inhibited from binding to the IR promoter by a phosphorylation catalyzed by PKCε [8], [14].

In Alzheimer's disease (AD), PKCε activators, cyclopropanated fatty acid derivatives DCP-LA and DHA-CP6, have been found to reduce amyloid β levels by enhancing the degradation of amyloid precursor protein (APP) [15], whereas overexpression of APP in turn decreases the levels of both membrane-bound active PKCε and cytosolic inactive PKCε in three different cell lines [16]. Moreover, overexpression of constitutively active PKCε leads to increased secretion of the neuroprotective peptide sAPP, which is cleaved from APP by α-secretase [17]. Preliminary animal studies support the role of PKCε in Alzheimer's disease, since PKCε activation in a transgenic mouse strain containing familial AD mutations was found to prevent amyloid plaques, synaptic loss and cognitive deficits [18].

PKCε is considered a desirable drug target for the treatment of cancer, AD and diabetes among other diseases. However, since different PKC isozymes can have different or even opposing roles in the same process [19], any therapeutic agents would have to be PKCε isozyme specific in order to have the desired therapeutic effect. The group of Dr. Mochly-Rosen has described the identification and characterization of a PKCε translocation inhibitor (εV1-2) [20] and a PKCε agonist peptide (ψεRACK) derived from the PKCε RACK [21]. Furthermore, they have shown that other peptides derived from the C2 domain of PKCε have the potential to act as PKCε agonists or antagonists [22].

We have previously reported the selection and screening of another class of PKCε specific activators and inhibitors, namely VHHs [23]. VHHs are the antigen binding regions of llama single chain antibodies that contain three complementary determining regions (CDRs) involved in antigen binding [24]. VHHs are highly soluble and stable, antigen-specific, and easy to produce [25]. They tend to have nanomolar affinities to their target antigens, and VHHs with affinities even in the picomolar range have been described [24]. Due to their unique structure, VHHs can also recognize conformational epitopes such as enzyme active sites that cannot be recognized by conventional antibodies. Furthermore, especially the long CRD3 loops of VHHs could serve as perfect leads for the design of new peptide drugs against various enzymes [25]. These advantages of VHHs compared to conventional antibodies, together with the positive data from the first clinical trials carried out with VHHs, indicate that VHHs are promising therapeutics, which will undoubtedly contribute to medicine in the future [26].

Here we report further details of the PKCε specific VHH activators (A10, C1 and D1) and inhibitors (E6 and G8) described previously [23]. Based on surface plasmon resonance (SPR) studies, the three activators and two inhibitors have affinities in the micromolar range. Furthermore, we show that the VHHs display species specificity since they do not bind the rat PKCε despite the 98% identity between the human and rat proteins. These VHHs were also tested in kinase activity assays to determine the Michaelis-Menten kinetics of activation or inhibition. Finally, we show that the VHHs have an effect on PKCε activity in a cellular context, since the activator A10 increases both the rate and degree of PKCε translocation in response to PMA stimulation in HeLa cells, whereas the inhibitor G8 slows down PKCε translocation. The results presented here give insight into the mechanisms of PKCε activation or inhibition by VHHs and highlight the conformation specific nature of the binding between these VHHs and their target protein. Moreover, the results demonstrate that these VHHs expressed inside HeLa cells as intrabodies have the ability to influence PKCε translocation, a step that is required for PKCε activation.

## Results

### Affinity measurements using Surface Plasmon Resonance

We have already shown by immunoprecipitation (IP) and kinase activity assays that the VHH activators and inhibitors of PKCε bind the human PKCε protein [23]. In the present study we further characterized the VHHs by determining their affinities to PKCε. Therefore, affinity measurements with surface plasmon resonance (SPR) technology were performed. First, we tried to determine the affinities using Biacore SPR technology (GE Healthcare, UK), which is commonly used to study the interactions of VHHs and their antigens [27]–[29]. A CM5 chip was coated with human recombinant PKCε using standard amino-coupling, and binding of VHHs to PKCε was studied in a Biacore T100 instrument. None of the tested VHHs bound to PKCε in this setup (data not shown). Next, each VHH was amino-coupled to the surface of a CM5 chip and the binding of PKCε to the flow cell surface was studied. This setup also failed, since PKCε bound to the surface of the reference flow cell as well as the VHH-coated flow cells (data not shown). These results were in strong contrast to the enzyme-linked immunosorbent assay (ELISA) and IP results described previously [23].

**Figure 1:**
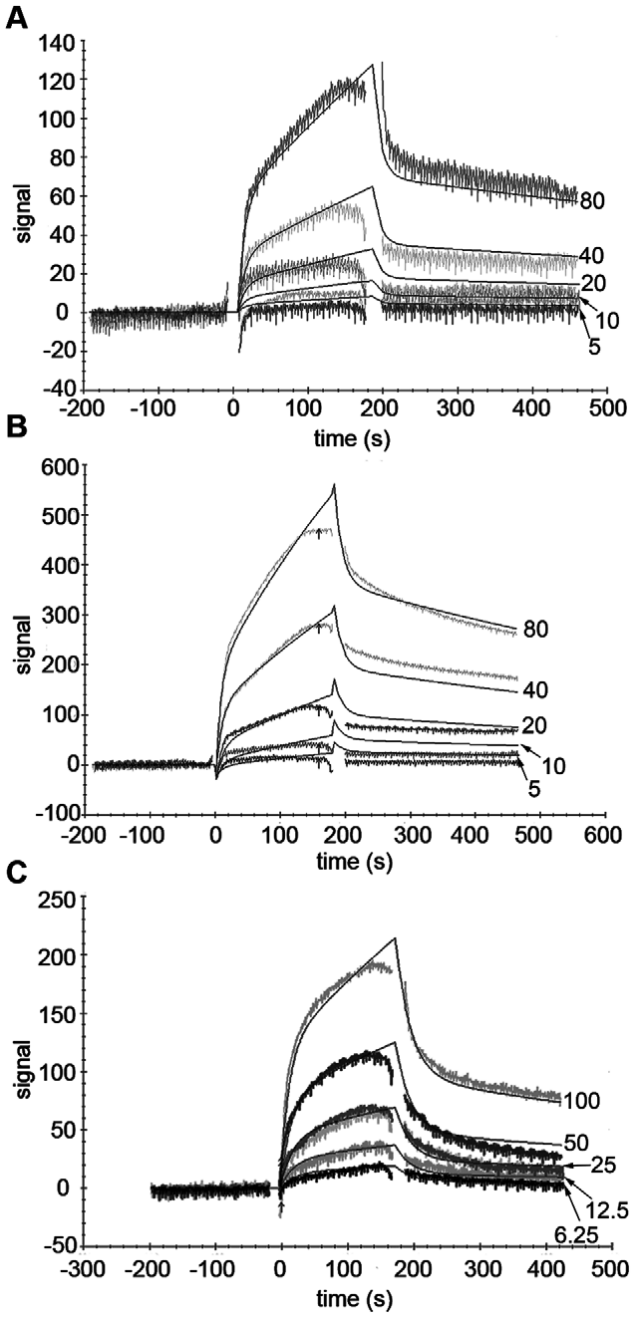
SPR sensograms and fits for PKCε activating VHHs. SPR sensograms and fits for second-order Langmuir binding models are shown for VHHs A10 (A), C1 (B) and D1 (C). The VHH injection time was 3 min, followed by a dissociation time of 5 min. The surface was regenerated with an injection of 10 mM NaOH for 3 min, followed by a stabilization time of 5 min between each VHH injection. Five concentrations of each VHH were used, with the middle concentration injected twice as an internal control. The VHH concentrations (in µg/ml) are marked adjacent to each fit on the right hand side of the figure.

**Figure 2:**
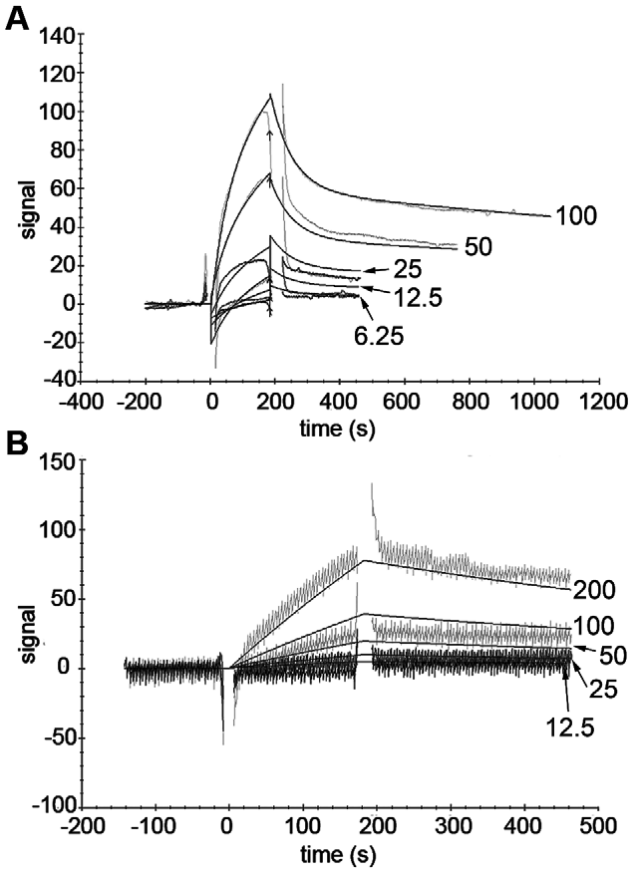
SPR sensograms and fits for PKCε inhibiting VHHs. SPR sensograms and fits for a second-order Langmuir binding model of VHH E6 (A) and a first-order Langmuir binding model of VHH G8 (B). The VHH injection time was 3 min, followed by a dissociation time of 5 min. The surface was regenerated with an injection of 10 mM NaOH for 3 min, followed by a stabilization time of 5 min between each VHH injection. Five concentrations of each VHH were used, with the middle concentration injected twice as an internal control. The VHH concentrations (in µg/ml) are marked adjacent to each fit on the right hand side of the figure.

The Bionavis SPR Navi 200-equipment was then used to study binding affinities. When PKCε was amino-coupled to the surface of the flow cell, none of the VHHs showed binding to PKCε, as was the case with Biacore. However, when the dextran hydrogel was amine-functionalized using ethylene diamine and PKCε was carboxyl-coupled to the surface of the flow cell, VHH binding to PKCε was detected (figures 1 and 2). The VHHs were injected in serial dilutions with five different concentrations for every VHH. The middle concentration was injected twice and served as an internal control. The resulting data was analyzed with TraceDrawer 1.3 from Bionavis.

The binding of VHHs A10, C1, D1 and E6 to PKCε was best fitted with second order Langmuir binding models based on the forms of the binding curves. Therefore, two association constants (k_a_ values), two dissociation constants (k_d_ values) and two affinities (K_D_ values) were calculated for each of these VHHs. The most likely explanation for the second order models is that PKCε was present in two or more different conformations on the surface of the flow cell, and binding of the VHHs to two of these conformations, with the strongest interaction affinities to VHHs, could be detected. An alternative explanation is that the VHH samples contained two different proteins that bound PKCε, but this is highly unlikely since the purity of the VHH samples was always checked on a Coomassie stained protein gel and found to be over 95% (data not shown). The G8 data was fitted with a first order Langmuir model, resulting in single k_a_, k_d_ and K_D_ values for this VHH.

Out of the activators (A10, C1 and D1), C1 had the highest affinities for PKCε coupled to the surface of the flow cell (figure 1B and table 1), namely 3.38 µM and 7.3 µM. D1 had affinities of 44.2 µM and 7.91 µM (figure 1C and table 1) and ranked second in affinity among the PKCε activating VHHs. A10 had the lowest affinities of the three activators (25.4 µM and 104 µM; figure 1A and table 1). In kinase activity assays, C1 caused the greatest increase in PKCε activity, followed by D1 and A10 [23]. Since C1 had both the highest affinity of the three activators and led to the greatest increase in PKCε activity, followed by D1 and A10, the affinities measured here for the three activators support the results from kinase activity assays.

**Table 1:** Association and dissociation constants for the interaction of VHHs with PKCε obtained from SPR measurements.

VHH	k_a_ 1 (1/(M*s))	k_d_ 1 (1/s)	K_D_ 1 (M)	k_a_ 2 (1/(M*s))	k_d_ 2 (1/s)	K_D_ 2 (M)
**A10**	2.95×10^1^ (±4.31×10^2^)	7.50×10^−4^ (±7.62×10^−5^)	2.54×10^−5^ (±1.76×10^−6^)	1.01×10^3^ (±1.98×10^3^)	1.05×10^−1^ (±3.27×10^−6^)	1.04E×10^−4^ (±7.21×10^−5^)
**C1**	3.09×10^2^ (±1.60×10^2^)	1.04×10^−3^ (±3.56×10^−5^)	3.38×10^−6^ (±2.56×10^−6^)	1.12×10^4^ (±4.11×10^3^)	8.21×10^−2^ (±4.10×10^−6^)	7.30×10^−6^ (±3.08×10^−6^)
**D1**	2.41×10^1^ (±2.89×10^−1^)	1.07×10^−3^ (±2.00×10^−4^)	4.42×10^−5^ (±8.80×10^−6^)	5.51×10^3^ (±1.27)	4.35×10^−2^ (±9.02×10^−5^)	7.91×10^−6^ (±1.82×10^−8^)
**E6**	5.22×10^2^ (±2.30×10^2^)	3.06×10^−4^ (±6.60×10^−5^)	5.87×10^−7^ (±4.78×10^−7^)	1.53×10^3^ (±4.59×10^3^)	1.49×10^−2^ (±5.10×10^−6^)	9.71×10^−6^ (±3.64×10^−6^)
**G8**	1.11×10^1^ (±7.11×10^2^)	1.13×10^−3^ (±4.01×10^−5^)	1.02×10^−4^ (±1.58×10^−6^)	n/a	n/a	n/a

Of the two inhibitors (E6 and G8), E6 (figure 2A) was a better binder of PKCε immobilized to the flow cell surface than G8 (figure 2B). The affinities of E6 to PKCε in this setup were 587 nM and 9.71 µM, whereas the K_D_ value for G8 was calculated to be 102 µM (table 1). As was the case with the activators, the obtained affinity constants support the results from kinase activity assays, where E6 is a more potent inhibitor of PKCε than G8 [23].

### Species specificity of PKCε activating and inhibiting VHHs

**Figure 3:**
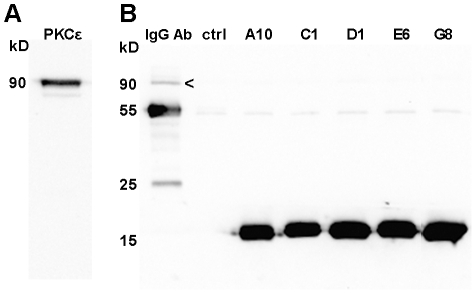
PKCε in rat brain extract. (A) 15 µg of rat brain extract was separated on a SDS-PAGE gel. PKCε was detected with anti-PKCε and HRP-conjugated goat anti-mouse antibodies. (B) Immunoprecipitations were performed with rat brain extract using a commercial anti-PKCε antibody (IgG Ab) and VHHs. PKCε (marked with an arrowhead) is visible at 90 kDa on lane 1. The bands at 55 kDa and 25 kDa on lane 1 represent the heavy and light chains of the anti-PKCε antibody. The bands at 16 kDa for A10, C1, D1, E6 and G8 represent the VHHs. A sample of uncoated protein A sepharose beads was included as a negative control (lane 2 = ctrl).

We have previously shown that VHHs A10, C1 and D1 increase human recombinant PKCε kinase activity, whereas VHHs E6 and G8 decrease kinase activity [23]. Rat brain extract is often used as an alternative source of PKC for experiments such as kinase activity assays, since it is known to contain many of the PKC isozymes, including PKCε [30], [31]. However, when kinase activity assays with the VHH activators and inhibitors of PKCε were performed using rat brain extract, no effect on kinase activity was seen (data not shown), even though based on Western blotting PKCε was present in the rat brain extract (figure 3A). The most likely explanation for this is that the VHHs do not bind the rat PKCε protein.

All of the VHHs that have been shown to have an effect on human PKCε kinase activity are able to immunoprecipitate human recombinant PKCε from Sf9 cell lysate [23]. To test whether the VHHs can also bind the rat PKCε protein despite the fact that they cannot influence its kinase activity, IPs were performed with rat brain extract. In addition, an IP with a commercial anti-PKCε antibody known to bind the rat form of the protein was included as a control. The five VHHs and the commercial anti-PKCε antibody were successfully captured by protein A beads (figure 3B). However, rat PKCε was only immunoprecipitated by the commercial anti-PKCε antibody and not by any of the VHHs. Therefore, the VHH activators and inhibitors of PKCε do not bind the rat PKCε protein, and hence cannot have an effect on its kinase activity. These results suggest that the VHHs are species-specific towards human PKCε, and confirm the very high specificity of the VHHs to human PKCε versus other PKC isozymes, an issue which could be a concern with peptide and other small molecule activators or inhibitors.

### Kinetic measurements of PKCε activation and inhibition

To characterize the kinetics of PKCε activation or inhibition by VHHs, kinase activity assays were performed with varying concentrations of the substrate peptide. The VHH concentration was kept constant (1 µg/well) for each experiment. When substrate concentrations are varied, the resulting data can be used to calculate the Michaelis-Menten kinetics of the activation or inhibition.

**Figure 4:**
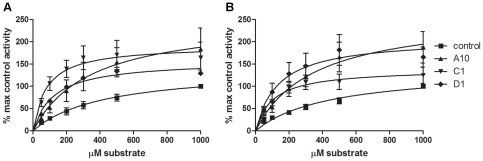
Kinetics of PKCε activation by VHHs A10, C1 and D1. The kinase activity of full-length PKCε in the presence (A) and absence (B) of PKC activators DOG and PS was measured with varying MARCKS substrate concentrations. The VHH concentration was constant (1 µg/well) for each experiment. The data is presented as percentage maximal control activity (control activity with 1000 µM substrate) ± SEM and represents at least 3 independent experiments, each with duplicates. Note that the V_max_ values for the VHHs have not been reached yet, see table 2 for analysis.

Results from the PKCε kinase activity assay with PKCε activators PS and 1,2-dioctanoyl-*sn*-glycerol (DOG; a DAG analogue) show that the three VHHs that act as PKCε activators have different mechanisms of activation (figure 4A and table 2). VHH A10 leads to increased PKCε activation by almost doubling the V_max_ value, or the maximum rate achieved by the system (141 nmol/min/mg for control and 253 nmol/min/mg for A10), whereas it has almost no effect on the K_m_ value of the reaction (figure 4A). In contrast, VHHs C1 and D1 have a much smaller effect on the V_max_, but they decrease the K_m_ value of the system from 424 µM for the control, to 81 µM for C1 and 126 µM for D1. A lower K_m_ value indicates that the reaction is faster relative to the V_max_, so C1 and D1 seem to increase the speed of the reaction instead of the maximum rate of the reaction. Since the K_m_ value is influenced both by the affinity of the enzyme to the substrate and the rate at which the substrate bound to the enzyme is converted to the product, the lower K_m_ value measured with VHHs C1 and D1 could indicate either an increase in the affinity or the rate at which the substrate is converted to the product.

**Table 2:** K_m_ and V_max_ values for PKCε activating and inhibiting VHHs.

	PKCε with activators PS and DOG (n≥3)		PKCε without activators PS and DOG (n≥3)		Catalytic domain (n≥2)	
	K_m_ (µM)	V_max_	K_m_ (µM)	V_max_	K_m_ (µM)	V_max_
**Control**	424	141	449	139	130	120
**A10**	348	253	352	263	no effect	no effect
**C1**	81	191	105	139	no effect	no effect
**D1**	126	158	137	208	no effect	no effect
**E6**	17	29	5.1	24	n/a	6.7
**G8**	260	113	121	71	158	43

The results for the activators were similar for the kinase activity assay that was performed with full-length PKCε without activators PS and DOG (figure 4B). Since VHHs A10, C1 and D1 have no effect or a very small effect on the activity of the catalytic domain alone [23], the Michaelis-Menten kinetics of the activators on the catalytic domain were not determined.

**Figure 5:**
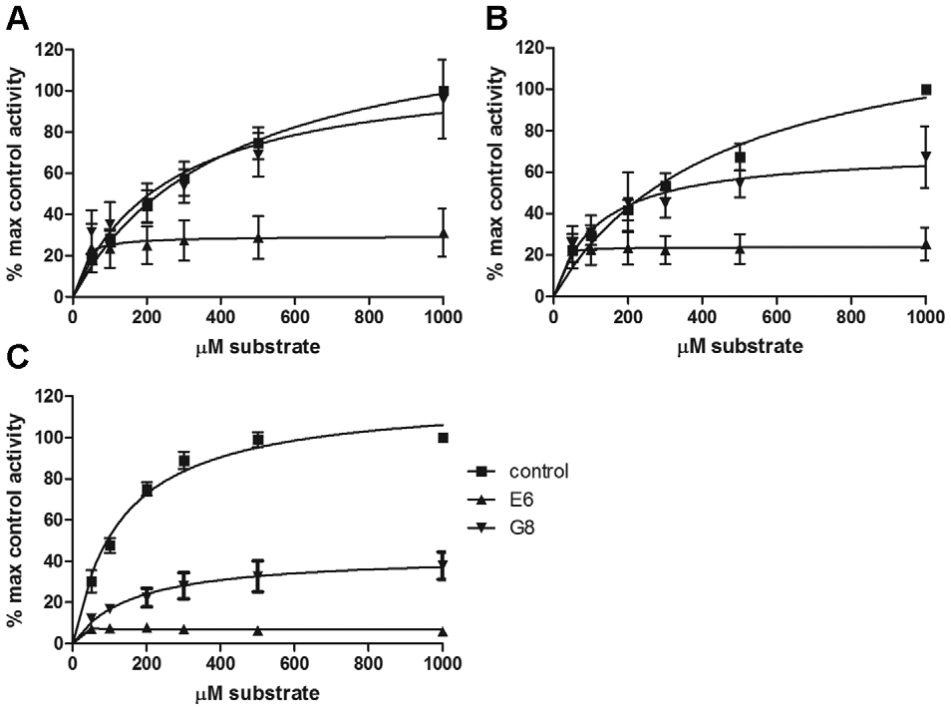
Kinetics of PKCε inhibition by VHHs E6 and G8. (A–B) The kinase activity of full-length PKCε in the presence (A) and absence (B) of PKC activators DOG and PS was measured with varying MARCKS substrate concentrations. (C) The kinase activity of the catalytic domain of PKCε was measured with varying MARCKS substrate concentrations. The VHH concentration was constant (1 µg/well) for each experiment. The data is presented as percentage maximal control activity (control activity with 1000 µM substrate) ± SEM and represents at least 3 independent experiments, each with duplicates. The catalytic domain activity (C) with G8 is an exception with only 2 independent experiments with duplicates.

Based on the Michaelis-Menten constants obtained for the two PKCε inhibiting VHHs E6 and G8, VHH E6 is a more efficient inhibitor of PKCε than G8 is. In the assay using the full-length PKCε protein with the activators DOG and PS present (figure 5A and table 2), E6 decreases the V_max_ from 141 nmol/min/mg (control) to 29 nmol/min/mg, whereas G8 leads to a more moderate decrease (V_max_ of 113 nmol/min/mg).

When the PKCε activators DOG and PS are not included in the assay (figure 5B), the difference between E6 and G8 is less and even G8 decreases the V_max_ by almost half. When the catalytic domain of PKCε is used instead of the full-length protein (figure 5C), the inhibition of kinase activity by E6 is so great that the K_m_ value cannot be reliably measured. In this case, E6 decreases the V_max_ from 120 nmol/min/mg to 6.7 nmol/min/mg. G8 is also a more potent inhibitor of the catalytic domain than the full-length protein, since it decreases the V_max_ of the reaction almost 3-fold.

### Analysis of PKCε inhibition by VHHs E6 and G8

The mechanism of PKCε inhibition by VHHs E6 and G8 was studied with kinase activity assays with varying substrate and VHH concentrations. We have previously shown that the binding site of both E6 and G8 is in the catalytic domain of PKCε [23]. Therefore, Sf9 lysate expressing the catalytic domain of PKCε was used for these assays.

**Figure 6:**
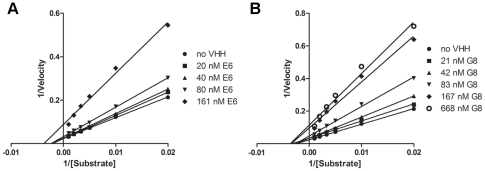
E6 and G8 are non-competitive inhibitors of PKCε. The activity of the catalytic domain of PKCε was measured with varying MARCKS substrate concentrations and varying concentrations of VHHs E6 (A) and G8 (B). The data was analyzed using non-linear regression and the Michaelis-Menten kinetics model and represents 3 independent experiments, each with duplicates. The data is presented as a Lineweaver-Burk plot to allow for the easy visualization of K_m_ and V_max_ values.

The data from these assays was analyzed using non-linear regression models but is represented as a Lineweaver-Burk plot to allow for easy visualization of the K_m_ and V_max_ values. As can be seen from figure 6, with both VHHs the K_m_ of the reaction remains about the same when the VHH concentration increases. However, the V_max_ decreases as the VHH E6 or G8 concentration increases. In the controls without VHH, the V_max_ is 46.7 nmol/min/mg, whereas at the highest VHH concentrations used in this experiment, the V_max_ is only 17.0 nmol/min/mg for E6 (figure 6A) and 15.9 nmol/min/mg for G8 (figure 6B).

According to the Michaelis-Menten kinetics, when the apparent K_m_ remains about the same but the V_max_ decreases with increasing inhibitor concentrations, the inhibition is non-competitive [32]. Therefore, E6 and G8 appear to be non-competitive inhibitors of PKCε that do not compete with the substrate peptide MARCKS for binding to PKCε.

### Effect of activator A10 and inhibitor G8 on PKCε translocation

PKCε is known to translocate to the cell membrane in response to PMA stimulation [3], [33]. In order to study whether the VHHs have an effect on the translocation of PKCε, one of the activating VHHs (A10) and one of the inhibiting VHHs (G8) were cloned to a mammalian expression vector and a C-terminal mCherry-tag was introduced to the sequence. HeLa cells were then double-transfected with PKCε-EGFP and the A10-mCherry or G8-mCherry plasmids, or an mCherry control plasmid. Translocation studies were performed with a confocal microscope 24 hours after transfections by stimulating cells with 100 nM PMA and monitoring the cellular localization of PKCε-EGFP and mCherry constructs for 30 minutes.

**Figure 7:**
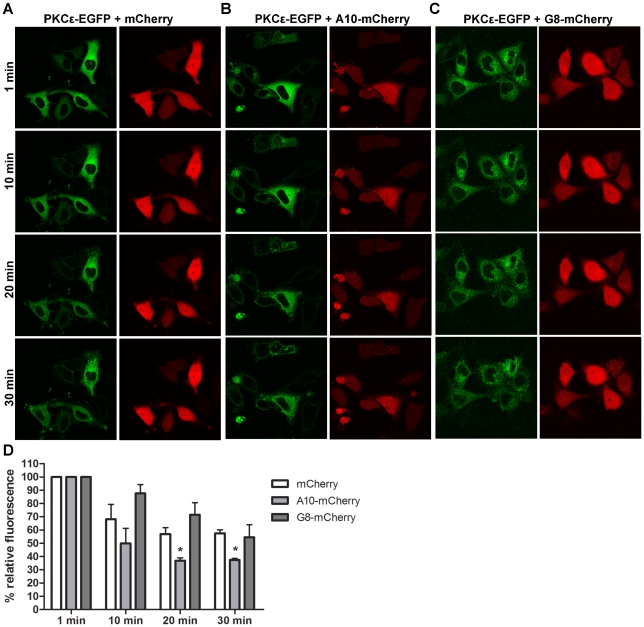
Activator A10 increases and inhibitor G8 decreases the rate of PMA-induced PKCε-EGFP translocation in HeLa cells. (A–C) Representative images of HeLa cells transfected with PKCε-EGFP and mCherry (A), A10-mCherry (B) or G8-mCherry (C) taken with a confocal microscope at 1, 10, 20 and 30 minutes after adding 100 nM PMA. (D) Quantification of PKCε-EGFP translocation from the cytoplasm over time. Data is presented as percentage relative fluorescence in the cytoplasm of cells ± SEM from at least 2 independent experiments with 4–6 cells per experiment (mCherry n = 4, A10-mCherry n = 3, G8-mCherry n = 2). The difference between cells transfected with the mCherry control plasmid and cells transfected with A10-mCherry was statistically significant (p<0.05) at 20 and 30 minutes (denoted with *).

In cells transfected with the mCherry control plasmid, about 70% of PKCε-EGFP remained in the cytoplasm 10 minutes after PMA stimulation (figure 7A and D). Strikingly, in cells transfected with the PKCε activator A10-mCherry, only 50% of PKCε-EGFP was still present in the cytoplasm at this time point (figure 7B and D). In contrast, in cells transfected with the PKCε inhibitor G8-mCherry, 90% of PKCε-EGFP was still present in the cytoplasm of the cells 10 minutes after PMA stimulation (figure 7C–D).

After 20 minutes, about 55% of PKCε-EGFP was present in the cytoplasm in mCherry transfected control cells. The amount of green fluorescence remained constant in the cytoplasm after this time point (figure 7A and D). In cells transfected with A10-mCherry, only about 40% of PKCε-EGFP was present in the cytoplasm 20 minutes after PMA stimulation. As was the case with the mCherry control transfected cells, the amount of fluorescence in the cytoplasm remained at the same level from 20 to 30 minutes in A10-mCherry transfected cells (figure 7B and D). In cells transfected with G8-mCherry, 70% of PKCε-EGFP remained in the cytoplasm of the cells at 20 minutes after PMA stimulation. In these cells, more PKCε-EGFP translocated to the membranes during the last 10 minutes of the experiments, since at the end of 30 minutes around 55% of PKCε-EGFP remained in the cytoplasm of G8-mCherry transfected cells (figure 7C–D). Even though a clear difference in translocation speed could be seen between mCherry transfected control cells and cells transfected with the PKCε inhibitor G8-mCherry, this difference did not reach statistical significance at any of the time points. The difference in PKCε-EGFP translocation between control cells transfected with mCherry and A10-mCherry transfected cells reached statistical significance at 20 and 30 minutes after PMA stimulation. Therefore, the PKCε activator A10 increases both the rate and the extent of PMA-induced PKCε translocation in HeLa cells, whereas the inhibitor G8 slows down the rate of PKCε translocation from the cytoplasm to the membranes.

## Discussion

VHH antibodies generally have affinities comparable to those of conventional antibody fragments, with K_D_ values in the nanomolar range [25], and VHHs with affinity constants as low as 100 pM have been described [24]. The high affinities displayed by most VHHs are one of the main advantages of VHH antibodies in research and drug development. However, here we report affinity constants for PKCε activating and inhibiting VHHs ranging from 587 nM to 104 µM.

One reason for the relatively low affinities described here could be the fact that only one round of VHH selections was carried out to obtain PKCε binders [23]. However, selections were done from an immune VHH library and usually antigen affinities of VHHs from immunized libraries are 10–100 times better than the affinities of VHHs isolated from naïve or synthetic libraries [25].

Another factor that probably contributes to the relatively low K_D_ values is the fact that these VHHs can only bind the native form of PKCε. The VHHs can bind human PKCε in immunoprecipitations and kinase activity assays, but not in Western blots where the PKCε protein has been denatured [23]. Furthermore, the VHHs tested here show relatively weak binding in VHH ELISAs (Summanen *et al.*, unpublished results), where PKCε has been coated on the wells of 96-well plates.

Assays such as ELISA and SPR, where the antigen has to be immobilized on a surface in order to measure an interaction, can be problematic when conformation dependent interactions are studied [34]. When the protein is immobilized using functional groups such as −NH_2_ or −COOH groups, the protein molecules are randomly oriented on the surface [35]. Therefore, only some of the protein molecules will be present in an orientation that can be recognized by the interaction partner, in this case the VHHs. Furthermore, when the interaction between randomly immobilized protein and the surface is too strong, there is a possibility of protein denaturation [35].

Since the affinities of the five VHHs studied here could not be measured at all with Biacore or Bionavis SPR when PKCε was amino-coupled to the chip, it is clear that the orientation of PKCε in the chip is crucial for measuring binding. Binding was observed when PKCε was carboxyl-coupled instead, but also in this case only some of the coated PKCε molecules would have been in the correct orientation and conformation. The critical role of protein orientation in SPR measurements is emphasized by the finding that oriented immobilization of an antibody increased its immunobinding efficacy approximately two-fold compared to standard amino-coupling [36]. Therefore, the best option would have been to immobilize PKCε to the flow cell in a controlled orientation via for example a His-tag [35], [37], but due to technical restrictions we were not able to perform such measurements.

As the interaction between the VHHs and PKCε seems to be conformation dependent, the affinity constants measured here are not likely to be the absolute affinities of these VHHs to PKCε in the solution phase. The reported K_D_ values should therefore not be compared to the affinities reported for VHH antibodies elsewhere. However, the obtained affinity constants can be used for internal comparison to determine which PKCε binders display the strongest interaction to PKCε. The scientific value of the K_D_'s reported here is evident from the fact that the affinities do support the data obtained from other experimental setups. Particularly, VHH C1, the strongest activator of PKCε, also has the strongest affinity for PKCε among the three activating VHHs. Furthermore, E6, which is a more potent inhibitor of PKCε kinase activity than G8, also has a higher affinity for PKCε than G8.

We also showed that in addition to the VHHs being conformation dependent, they seem to be species-specific as well. While all five VHHs bind human PKCε and either increase or decrease its kinase activity, they have no effect on the kinase activity of rat PKCε, nor did they bind it in an IP. Both human and rat (*Rattus norwegicus*) PKCε proteins are 737 amino acids in length, and identical for 726 of these amino acids (98%). Within the catalytic domain, where all of the VHHs described here bind to, the human and rat proteins differ in only eight amino acids. One would expect PKCε specific antibodies to bind to both proteins since the differences between them are so small. However, as reported here, the VHHs do not bind rat PKCε, which again demonstrates how subtle differences in amino acid composition or protein conformation play a critical role in the binding of these PKCε activating and inhibiting VHHs. Further mapping of the VHH binding site will show if the observed species specificity is due to a specific amino acid substitution or a small conformational difference between the human and rat proteins.

The kinetics of the PKCε activation and inhibition were also studied in more detail. We show here that the three activators A10, C1 and D1 increase PKCε activity in different ways. A10 nearly doubles the maximum rate of the reaction, whereas C1 and D1 have almost no effect on the V_max_ but increase the speed of the reaction relative to the V_max_, as is evident from the smaller K_m_ values reported for these VHHs. These results were similar with and without the PKC activators DOG and PS present in the assay. We know from previous studies [23] that all of the activating VHHs bind the catalytic domain of PKCε. Since the VHHs can increase PKCε kinase activity in an *in vitro* assay without any additional proteins present, it seems likely that the VHH binding somehow stabilizes the active conformation of PKCε. In order to determine the method of PKCε activation for each VHH, the exact binding sites for each VHH must be studied.

There are also differences between the two PKCε inhibiting VHHs E6 and G8. Based on the kinase activity assay results reported here, E6 is a more potent inhibitor of PKCε, since it leads to a larger decrease in the maximum rate of the reaction. With full-length PKCε, the V_max_ is only around 20% of the control with E6, whereas G8 has a much smaller effect on full-length PKCε activity. When the catalytic domain is used instead of the full-length protein, G8 also displays a larger degree of inhibition. This supports previous results [23], where G8 was found to be a better inhibitor of the catalytic domain alone than the full-length protein, possibly because in the full-length protein, the G8 binding site could be partially concealed.

According to Michaelis-Menten kinetics, both E6 and G8 are non-competitive inhibitors of PKCε, since increasing VHH concentrations had no effect on the apparent K_m_ of the system but demonstrated clear decreases in V_max_. Therefore, we can rule out the substrate-binding site from the possible binding sites of E6 and G8 within the catalytic domain of PKCε. However, as is the case with the activating VHHs, there are several possible mechanisms by which E6 and G8 can have an effect on PKCε kinase activity. A more detailed explanation of PKCε inhibition warrants a study into the exact binding sites of E6 and G8 in the catalytic domain of PKCε.

Remarkably, we also demonstrated for the first time that the PKCε activating and inhibiting VHHs can influence PKCε activity when expressed inside HeLa cells. Upon PMA stimulation, PKCε translocates from the cytoplasm to the plasma membrane, as was shown with EGFP-tagged PKCε in HeLa cells. The activating VHH A10 expressed inside HeLa cells with a C-terminal mCherry-tag increased both the rate and the degree of PKCε translocation compared to the control. On the other hand, the inhibiting VHH G8 decreased the rate of PKCε translocation in response to PMA. Since PKCε translocation is required for activation, we can conclude that the VHHs can influence PKCε activity also in a cellular context. These results highlight the potential of activity modulating VHHs in PKCε research and drug development. Furthermore, the observed cellular effects suggest that the affinities of the VHHs to PKCε are in fact better than the micromolar affinities obtained from SPR experiments. Inside cells, both PKCε and the VHHs will be properly folded, allowing the VHHs to bind to their conformational epitopes on PKCε surface.

The results described here provide important additional information about the VHH activators and inhibitors of PKCε. In addition to the peptide-based PKCε agonists and translocation antagonists [20], [21], these VHHs are the only strictly PKCε isozyme specific activators and inhibitors described so far. Since the different PKC isozymes can have overlapping and sometimes even opposing roles in many biological processes, such isozyme specific compounds that influence kinase activity are crucial in studying the role of PKCε in various contexts. Furthermore, PKCε specific VHHs could in the future be developed into therapeutics against diseases such as cancer or type II diabetes, or the CDR regions of VHHs could be used to design novel peptide-based therapies against these life-threatening diseases.

## Materials and Methods

### Materials

Mercaptoundecanol, epichlorohydrin, dextran (500 kDa from *Leuconostoc* spp.), bromoacetic adid, N-ethyl-N′-(dimethylaminopropyl) carbodiimide (EDC), N-hydroxysuccinimide (NHS), phosphate buffered saline (PBS) tablets, and ethanoldiamine were all obtained from Sigma Aldrich (St. Louis, MO). Gold-coated SPR sensor slides were obtained from BioNavis Ltd (Tampere, Finland).

### Production and purification of VHHs

Monoclonal VHH antibodies were produced and purified as previously described [23]. Briefly, VHH production in *E. coli* JM109-strain was induced by the addition of 1 mM isopropyl β-D-1-thiogalactopyranoside (IPTG) overnight at 30°C. Periplasmic fractions were prepared by freezing the bacterial cell pellets for 1 h at −80°C to break the outer membrane of *E. coli* and resuspending cells in 10 ml of phosphate buffered saline (PBS), followed by mixing for 2 h at 4°C. VHHs were purified from the periplasmic fraction using the his-tag and Talon Metal Affinity Resin (Clontech, CA) and eluted with 300 mM imidazole. Eluted VHHs were dialysed against PBS overnight at 4°C and stored at −20°C until used.

### Expression of PKCε in Sf9 cells

Human full-length PKCε and its catalytic domain (amino acids 298–737) were produced in Sf9 cells using the baculovirus expression system (Bac-to-Bac, Invitrogen, Carlsbad, CA). The cloning of PKCε constructs and baculovirus stock production has been described before [23]. For expression of recombinant PKCε, Sf9 cells were infected with an optimized amount of baculovirus stock and grown for 48 h at 27°C in suspension. The collected cells were washed with PBS and frozen until used. Crude cell lysates were prepared by resuspending cells in lysis buffer containing 25 mM Tris-HCl, pH 7.5, 0.5 mM EGTA and 0.1% Triton X-100, supplemented with a protease inhibitor cocktail (Complete, Roche, Basel, Switzerland) and centrifuging for 15 min at 4°C at 16.200 g. Protein concentrations of the supernatants were determined by Bradford assay (Sigma-Aldrich, St. Louis, MO) and used for kinase activity assays as described below.

### Surface Plasmon Resonance

The affinity measurements were performed with BioNavis SPR Navi 200 (BioNavis Ltd, Tampere, Finland). Carboxymethylated dextran hydrogel for ligand immobilization was self-synthesized according to the BioNavis protocol. First a self-assembled monolayer of mercaptoundecanol was formed on clean gold-coated SPR sensor slides in an overnight reaction in ethanol and rinsed thoroughly. The sensor was then left to react for 3 h with epichlorohydrin (2% v/v) in 0.1 M NaOH, whereafter it was rinsed with Milli-Q H_2_O, transferred to 30 g/l solution of dextran in 0.1 M NaOH and left to react for 24 h. After washing thoroughly with Milli-Q H_2_O the sensor was immersed in 0.5 M bromoacetic acid in 2 M NaOH for 24 h. After this reaction the sensor was thoroughly washed with Milli-Q H_2_O and stored at +8°C until used in protein immobilization reaction.

Protein immobilization to the hydrogel was performed with reverse activated ester synthesis according to the BioNavis protocol. In brief, the immobilization was performed *in situ* in the instrument using Sigma's PBS (0.01 M phosphate buffer, 2.7 mM KCl and 137 mM NaCl, pH 7.4) as background and injection buffer. A flow rate of 20 µl/min and an injection time of 8 min was used for all injections. Reference surface was created in flow channel 2 in parallel with the protein immobilization. The channel was treated in exactly the same manner as the sample channel, except that instead of PKCε protein blank PBS was injected.

The flow cell surface was cleaned with an injection of a solution containing 2 M NaCl and 10 mM NaOH. Activation of the surface was performed by an injection of a solution consisting of 200 mM EDC and 50 mM NHS. Ethylene diamine (10 mg/ml) was injected in order to amine-functionalize the dextran hydrogel. PKCε was diluted to 10,5 µg/ml with the EDC/NHS activation solution, mixed well and immediately injected to the instrument. Protein immobilization of approximately 80 pg/mm^2^ was observed.

The experiments were performed in HBS (Hepes buffered saline; 20 mM Hepes, pH 7.5, 150 mM NaCl, 1 mM EDTA, 0.001% Tween-20) measurement buffer, with a temperature of 21°C and a flow rate of 20 µl/min with 8 min injection times. Serial dilutions of the VHHs (A10 and C1: 5 µg/ml to 80 µg/ml; D1 and E6: 6.25 µg/ml to 100 µg/ml; G8: 12.5 µg/ml to 200 µg/ml) were injected, as is required for kinetic analysis of molecular interactions [38]. NaOH (10 mM) was found to be an effective regeneration agent for the system, and was used as regeneration solution between each consecutive injection.

### Rat brain extract preparation

Two rats were asphyxiated with CO_2_ gas and then decapitated. The skulls were cut open and the brain tissue was scraped into ice cold PBS. The brain tissue was then homogenized with Dounce tissue homogenizer in buffer containing 10 mM HEPES pH 7.5 and 2 mM EDTA. The homogenized tissue was centrifuged at 1000 g for 10 min at 4°C. The resulting supernatant was centrifuged further for 1 h at 40 000 g at 4°C. The extract was then poured into an ion exchange column containing diethylaminoethyl cellulose (DEAE) in column equalization buffer (10 mM HEPES pH 7.5, 2 mM EGTA and 2 mM EDTA). The column containing the extract was extensively washed with column equalization buffer and the remaining bound proteins were subsequently eluted with buffer containing 10 mM HEPES pH 7.5, 2 mM EGTA, 2 mM EDTA, 200 mM NaCl, and 10 mM β-mercaptoethanol. The eluted protein fractions were combined and the protein content was determined. The brain extract was stored at −20°C after addition of 50% glycerol (final concentration).

### Western blots

To confirm the presence of PKCε in rat brain extract, 15 µg of protein from rat brain extract was separated by SDS-PAGE gels and blotted onto a PVDF-membrane. The blot was probed with 1∶1000 dilution of mouse anti-PKCε antibody (BD Biosciences, NJ), followed by a 1∶4000 dilution of HRP-conjugated goat anti-mouse antibody (Santa Cruz Biotechnology, CA).

### Immunoprecipitations of PKCε from rat brain extract

Immunoprecipitations (IPs) were done to check whether the VHHs can bind the rat PKCε protein. IPs were started by incubating 30 µl of Protein A sepharose CL-4B beads (GE Healthcare, United Kingdom) washed once with immunoprecipitation buffer (IPB; 25 mM Tris-HCl pH 7.5, 0.1% Triton X-100) with 3 µg of VHH in 1 ml of IPB for 1 h at 4°C with continuous shaking. Simultaneously, a sample of protein A sepharose was also coated with 1 µg of commercial PKCε antibody (BD Biosciences, NJ) in 1 ml IPB and was used as a positive control. The antibody-coated protein A beads were blocked with 1% BSA in IPB for 15 min at 4°C and washed once with IPB. Rat brain extract (200 µg/sample) was added to the beads and incubated overnight with continuous shaking at 4°C. The beads were washed 4 times with IPB and resuspended in 15 µl of 2× Laemmli sample buffer. Samples were loaded onto 15% SDS-PAGE gels for separation of proteins, electrotransferred to PDVF membranes and the membranes were then incubated with anti-PKCε (BD Biosciences, NJ) or anti-tetra His (Qiagen, Venlo, the Netherlands) antibodies followed by HRP-conjugated anti-mouse antibodies (Santa Cruz Biotechnology, CA).

### Kinase activity assays

Kinase activity assays were carried out as described before [23]. Briefly, kinase activity was determined by measuring the incorporation of [γ-^32^P] into a PKC substrate peptide MARCKS (FKKSFKL). For determining K_m_ and V_max_ values, 5 µg of protein from Sf9 cell lysate expressing full-length PKCε or PKCε catalytic domain was pre-incubated with 1 µg of VHH and the substrate (0 µM–1000 µM) in a total volume of 25 µl for 10 min at 30°C in a 96-well plate. Reaction mix (75 µl/well) was added, yielding final concentrations of 10 mM HEPES pH 7.5, 7 mM MgCl_2_, 0.25 mM EGTA, 100 µM cold ATP and 0.3 µM [γ-^32^P]ATP. Activity of the full-length PKCε was measured with and without PKC activators phosphatidylserine (40 µg/ml) and 1,2-dioctanoyl-*sn*-glycerol (DOG; 8 µg/ml). Kinase reactions were performed for 5 min at 30°C, after which 25 µl/well was pipetted to a P81 cation exchange paper (Whatman, Kent, United Kingdom). The papers were washed with 75 mM phosphoric acid, dried and placed in scintillation tubes with scintillation fluid. Radioactivity was measured by liquid scintillation counting (1414 Winspectral, Wallac, Finland). For the analysis of PKCε inhibition by E6 and G8, the assay was performed in the same way, except that only the catalytic domain of PKCε was used and VHH concentrations/well ranged from 20–161 nM (E6) and 21–668 nM (G8). Rat brain extract (1 µg/well) was used as an alternative source of PKCε.

### Cloning of VHH-mCherry constructs and purification of plasmid DNA

The activator A10 and the inhibitor G8 were cloned into the pcDNA3.1+ mammalian expression vector (Invitrogen, Carlsbad, CA) with a C-terminal mCherry tag. The mCherry plasmid was a generous gift from Prof. Roger Tsien (University of California, San Diego, CA). First, the mCherry-sequence was cloned into the pcDNA3.1+ vector using the *Bam*HI and *Eco*RI restriction sites. The resulting mCherry-pcDNA3.1+ plasmid was verified by sequencing.

The cDNA for the VHHs was PCR amplified from Pax50 bacterial expression vectors using the forward primer 5′-GGCGCTAGCATGGCAGAGGTGCAG-3′ and the reverse primer 5′-GGCAGATCTCCCGTGATGGTGATG-3′ to introduce the *Nhe*I and *Bgl*II restriction sites. The His_6_-tag that was on the C-terminus of the VHHs on the Pax50 expression vector was included in the cloning, so that the His_6_-tag is situated between the VHH and mCherry on the pcDNA3.1+ expression vector. The PCR amplified VHH fragments were digested with *Nhe*I and *Bgl*II and cloned into the mCherry-pcDNA3.1+ expression vector using the *Nhe*I and *Bam*HI sites (*Bgl*II and *Bam*HI have complementary sticky ends). The resulting VHH-His_6_-mCherry constructs were verified by sequencing and the plasmids were produced in the *E. coli* strain JM109.

Plasmid DNA for mammalian cell transfections was purified from E. coli cells using the PureYield™ Plasmid Midiprep System (Promega, Fitchburg, WI). To improve the purity of the eluted DNA, a subsequent ethanol precipitation step was performed and the dried DNA was diluted in TE buffer. The plasmid DNA was diluted to a concentration of 1 µg/µl and stored at −20°C. The PKCε-EGFP plasmid, which was a kind gift from Prof. Peter Parker (Cancer Research UK, London Research Institute), was produced and purified as described above.

### Cell culture

Human cervical cancer HeLa cells (CCL-2) were obtained from the American Type Culture Collection (ATCC, Manassas, VA) and cultured in DMEM supplemented with 10% fetal bovine serum (FBS). For transfections and treatments, DMEM without FBS was used. Cultures were incubated at 37°C in a humidified atmosphere of 5% carbon dioxide (CO_2_).

### HeLa cell transfections and translocation studies

For transfections, HeLa cells were seeded to 6-well plates (350 000 cells/well in 2 ml of FBS-supplemented DMEM) and incubated overnight to allow attachment. Double transfections of PKCε-EGFP and mCherry, A10-mCherry or G8-mCherry were carried out in serum-free medium with the FuGENE HD transfection reagent (Roche, Penzberg, Germany) according to the manufacturer's instructions. Translocation studies were performed at 37°C with a Leica SP2 AOBS confocal laser scanning microscope 24 hours after transfections. Double transfected cells expressing both fluorescent proteins were chosen for the experiments, and the 488 nm argon ion laser and the 561 nm He-Ne laser were used for the detection of EGFP-tagged PKCε and VHH-mCherry constructs, respectively. Typically the translocation of PKCε-EGFP was monitored in approximately 4–6 cells per experiment. Once double-transfected cells were located under the microscope, 100 nM PMA was carefully added to the cells. Images from the same cells were taken for 30 min every 30 sec. Translocation of PKCε-EGFP after PMA addition was quantified by measuring the relative fluorescence intensity in a region of interest with a diameter of 5 µm placed in the cytoplasm of each cell.

### Statistical analysis

SPR kinetic analysis of the results was performed with TraceDrawer 1.3 for BioNavis Ltd (Tampere, Finland). The measurements were double referenced, meaning that each sample was referenced using a blank reference channel on line and also 0-samples were measured and referenced from all sensograms during data analysis. Double referencing is a common procedure in SPR biosensor experiments [38]. The sensograms were fitted with either first order or, when appropriate, second order Langmuir binding models in the TraceDrawer software.

The data from kinase activity assays was analyzed and Michaelis-Menten kinetics were calculated with GraphPad Prism 4 (GraphPad Software Inc., La Jolla, CA) software using non-linear regression. The translocation of PKCε-EGFP in HeLa cells was quantified using Leica confocal LAS AF Lite software (Leica Microsystems, Wetzlar, Germany). The statistical significances in translocation speed in cells transfected with the mCherry and VHH-mCherry constructs were calculated with SPSS 15.0 software (SPSS Inc., Chicago, IL, USA) using a one-way Anova with Dunnett's post-test. Statistical significance was denoted with * when p<0.05.
